# Cooperative melting in double-stranded peptide chains through local mechanical interactions

**DOI:** 10.1098/rsif.2023.0130

**Published:** 2023-07-12

**Authors:** Luca Bellino, Giuseppe Florio, Alain Goriely, Giuseppe Puglisi

**Affiliations:** ^1^ Polytechnic University of Bari, Department of Civil Environmental Land Building Engineering and Chemistry (DICATECh), Via Orabona 4, Bari 70125, Italy; ^2^ INFN, Section of Bari, Bari 70126, Italy; ^3^ Mathematical Institute, University of Oxford, Oxford OX2 6GG, UK

**Keywords:** peptide chains, cooperativity, melting transition, local mechanical interactions

## Abstract

The separation of double-stranded peptide chains can occur in two ways: cooperatively or non-cooperatively. These two regimes can be driven either by chemical or thermal effects, or through non-local mechanical interactions. Here, we show explicitly that local mechanical interactions in biological systems may regulate the stability, the reversibility, and the cooperative/non-cooperative character of the debonding transition. We show that this transition is characterized by a single parameter depending on an internal length scale. Our theory describes a wide range of melting transitions found in biological systems such as protein secondary structures, microtubules and tau proteins, and DNA molecules. In these cases, the theory gives the critical force as a function of the chain length and its elastic properties. Our theoretical results provide quantitative predictions for known experimental effects that appear in different biological and biomedical fields.

## 1. Introduction

Polypeptide molecules such as DNA and proteins are the basic structural and information components in living systems. Their assembly and function are of paramount importance to avoid diseases and genetic defects. It is now appreciated that they operate not only through chemical and electrical effects, but also using mechanical and thermal fields [1–3]. Often, biomolecules undergo conformational transitions to perform tasks such as folding in proteins [4–6], DNA replication and denaturation [2,7,8], axonal growth [9] or focal adhesion mechanosensing [10–12]. The relative stability of different equilibrium states and their transition reversibility is particularly important for the associated biological functions.

Here, we focus on the cooperativity, stability and toughness of the mechanically induced melting transition in double-stranded molecules. *Cooperativity*, the emergent property of a transition based on the coordination of a network of interactions between different segments of the molecule, depends on the external field that drives the transition either through thermal [13] or chemical [14] effects. However, mechanical loading may lead to different responses, depending also on the properties of the system such as variations in linker stiffness, which can strongly decrease the transition cooperativity [15–17]. We show that, in a purely mechanical setting, cooperativity and stability effects can be induced by purely *local* mechanical interactions. These results are directly relevant for DNA and RNA hairpins [18] and to support the hypothesis of a zipper cooperativity model for protein folding [19,20], considered as a possible solution to Levinthal’s paradox.

Specifically, we consider a Peyrard–Bishop (PB) model composed of two chains of harmonic springs interacting through breakable shear links. Due to their simplicity that allows for analytic treatment, PB models played a key role to describe important phenomena related to the thermomechanical and dynamical behaviour of DNA [21–29], including melting studies depending on the ionic [30] and crowding environments [31]. This approach can also be extended to other double-stranded molecules, such as protein secondary structures or tropocollagen fibres, where the relation between the critical melting force and chain length is of paramount importance for the transition properties [32–35]. Moreover, PB models can be enriched to describe the temperature dependence of the melting transition, as recently reported in [36], where the case of the unzipping force of a DNA chain under orthogonal loading within the whole range of temperatures experimentally observed has been predicted. We argue that the possibility of quantitatively describing the cooperative→non-cooperative transition by varying the next-to-nearest interaction stiffness in the PB model supports once again the fundamental effect of local interactions. Other effects, such as torsional and bending stiffnesses or the evolution and nucleation of tertiary and quaternary structures in proteins, cannot be readily addressed by such models. However, a first possible extension in this direction is to introduce non-local effects [22,23,37,38].

Here, we obtain analytical solutions capturing crucial features such as the dependence of the rupture force on the length of the molecule, the persistence length, and the transition between cooperative and non-cooperative debonding strategies together with the corresponding transition energy.

In particular, for a chain of contour length *L*, we introduce a dimensionless parameter, the *cooperativity index* *μ* = *L*/\ell_*o*_. It depends on an emerging parameter, the *localization length* ℓ_*o*_, which generalizes the notion of persistence length for a single molecule and regulates the cooperativity and the melting energy. We select the value *μ* = 1 (*L* = ℓ_*o*_) such that if *μ* ≪ 1 the behaviour is cooperative and it is characterized by an all-or-none phenomenon, whereas if *μ* ≫ 1 the transition is non-cooperative, with a much higher ‘stability’ of partially debonded states. Interestingly, what emerges from our analysis is that cooperative transition can be obtained as a purely *local* mechanical effect. This completes previous models regulating cooperativity through *non-local* interactions [38–41].

Our model predicts important properties of biological systems where the cooperative/non-cooperative character of the melting transition is of crucial importance for their counterparts in physiology and medical fields. We derive analytical formulae describing the role of local mechanical interactions in the stability and the cooperativity of double-stranded molecules under external mechanical loading. In addition, we compare our theoretical results with experimental data and molecular dynamic simulations focusing on the phenomena of DNA denaturation, tropocollagen maximum pull-out force, and critical length of microtubules and tau proteins inside the neuronal axons.

## 2. Model

To analyse the main energetic components regulating the melting transition of a double-stranded molecule, we start from the classical PB model [39]. In particular, we consider two chains of *n* massless points connected by (identical) elastic springs with energy

**Figure 1.**
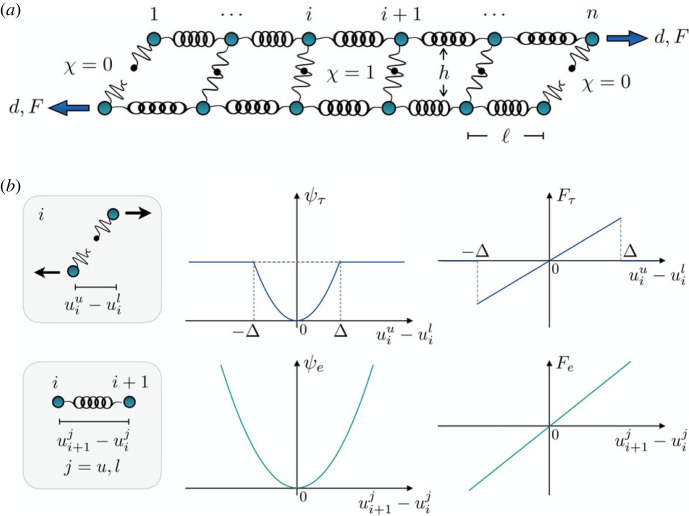
(*a*) Mechanical model describing a generic double-stranded molecule with breakable links connecting the elastic chains representing the strands. (*b*) Energy and force of a single breakable cross-link and of a single harmonic unit composing the strands.

2.1 $$\psi_{e}=\frac{1}{2}k_{e}\ell \sum_{\;j=up,low}\sum_{i=1}^{n-1}{\left(\frac{u_{i+1}^{j}-u_{i}^{j}}{\ell }\right)}^{2},$$

where ℓ is the spring length, *k*_*e*_ = *K*_*e*_ℓ measures the material stiffness, where *K*_*e*_ is the springs stiffness. The indices *up* and *low* indicate upper and low chains, respectively. The chains are connected by (identical) local breakable links (see figure 1), modelling non-covalent interactions such as H-bonds, and subjected to an endpoint displacement *d* with conjugated force *F*. Moreover, we assume that the shear links have an harmonic regime up to a displacement threshold Δ, followed by a zero force detached state. We note that, although re-cross-linking effects are typical under cyclic loading for biological systems, here we neglect them because we consider monotonic stretching.

To describe the state of the system, we introduce a ‘spin’ variable *χ*_*i*_ [42–44], with *χ*_*i*_ = 1 if $u_{i}^{up}-u_{i}^{low}<\Delta$, corresponding to attached states, and *χ*_*i*_ = 0 if $u_{i}^{up}-u_{i}^{low}\geq \Delta$ when the *i*th cross-link is broken. Accordingly, the interaction energy reads

2.2 $$\psi_{\tau }=\frac{1}{2}k_{\tau }\ell \sum_{i=1}^{n}\left[\chi_{i}{\left(\frac{u_{i}^{up}-u_{i}^{low}}{\Delta }\right)}^{2}+\;(1-\chi_{i})\right].$$

Here, *k*_*τ*_ = *K*_*τ*_ Δ^2^/*h* measures the material shear stiffness, where *K*_*τ*_ is the springs stiffness and *h* its length.

After introducing the non-dimensional displacements, force and energy

2.3 $$w_{i}=\frac{u_{i}}{\Delta },\;\delta =\frac{d}{\Delta },\;f=\frac{FL}{k_{e}\Delta }\;\mathrm{and}\;\varphi =\frac{\ell (\psi_{e}+\psi_{\tau })}{k_{e}\Delta^{2}},$$

at assigned displacement $w_{n}^{up}-w_{1}^{low}=2\delta$, we minimize the energy function

2.4 $$\min_{w_{i}^{up},w_{i}^{low}}\left[\varphi (w_{i}^{up},w_{i}^{low})-\frac{\;f}{n}(w_{n}^{up}-w_{1}^{low}-2\delta )\right],$$

where the force *f* represents a Lagrange multiplier. We remark that to focus on the mechanical behaviour here we neglect external thermal and chemical effects and minimize the total (elastic plus debonding) energy φ as in classical Griffith-type approaches to fracture and decohesion [45].

Equilibrium solutions obtained from (2.4) can be parametrized by the internal variables *χ*_*i*_. Indeed, the exact solution involves the inversion of a tri-diagonal Hessian matrix which depends on the configuration *χ*_*i*_ and is characterized by shears ($v_{i}=w_{i}^{up}-w_{i}^{low}$) attaining the maximum value at the system boundaries [46,47] (see electronic supplementary material). According to our assumption on (2.2), this implies that all local minimizers of the energy are characterized by a ‘*single-block'* of *p* attached elements. Due to the absence of non-local energy interactions, it is possible to show that all the solutions characterized by the same size of the attached/detached blocks (same number of *p*/*n* − *p* elements) are energetically invariant independently on the position of the remaining *n* − *p* detached elements that can be either in a single block or at the two endpoint blocks, possibly with different lengths. Conversely, interfacial energy terms could favour single interface solutions [48], as well as it is important to remark that when thermal effects cannot be neglected multi-block solutions can be observed with one or several ‘bubbles’ before melting. However, in many cases, DNA and proteins have been observed to debond through zipper fronts propagations, often single fronts [49,50]. Moreover, in [37], the authors present a numerical analysis supporting the hypothesis of single wall solutions at low values of temperatures even when thermal effects are introduced.

Under the single block hypothesis, the inverse of the matrix can be computed explicitly (see electronic supplementary material) and the equilibrium energy assumes the simple form

2.5 $$\varphi_{\mathrm{eq}}=\kappa^{t}\delta^{2}+\mu^{2}\left(1-\frac{\;p}{n}\right).$$

Correspondingly, the equilibrium force is given by

2.6 $$f=\kappa^{t}\delta ,$$

with

2.7 $$\kappa^{t}=\frac{4n}{\left(2n-p-1+4\gamma \right)}\;$$

being the chain stiffness, where

2.8 $$\gamma =\frac{\text{sinh}\lambda +\text{sinh}(p\lambda )}{2\{\text{sinh}[(p+1)\lambda ]-\text{sinh}\lambda -\text{sinh}(p\lambda )\}}$$

and

2.9 $$\lambda =\text{arccosh}\left(1+\frac{2\mu^{2}}{n^{2}}\right).$$

As previously anticipated, here we introduced the main non-dimensional parameter of the paper, i.e. the *cooperativity index* *μ*,

2.10 $$\mu^{2}=\frac{L^{2}}{\ell_{o}^{2}}=\frac{k_{\tau }}{2k_{e}}\frac{L^{2}}{\Delta^{2}},$$

where

2.11 $$\ell_{o}=\sqrt{\frac{2k_{e}}{k_{\tau }}}\Delta \;$$

is an internal length-scale measuring the *localization length* of the decohesion and plays a role similar to the persistence length for single chains [51]. These solutions (see electronic supplementary material) are defined for

2.12 $$\begin{array}{rl} & \delta \in [0,\delta_{\max}(p)],\;p=1,\ldots ,n,\\ & \;\delta_{\max}\;:=\frac{1}{4}\left\{\frac{\left(2n-p-1)\text{sinh}[(p+1)\lambda \right]}{\text{sinh}\lambda +\text{sinh}(p\lambda )}-2n+p+3\right\}. \end{array}$$

Here, based on previously recalled monotonicity results, *δ*_max_(*p*) is the maximum displacement inducing the cross-link *i* = *p* attaining the debonding threshold $u_{p}^{up}-u_{\;p}^{low}=\Delta$.

## 3. Melting strategies

**Figure 2.**
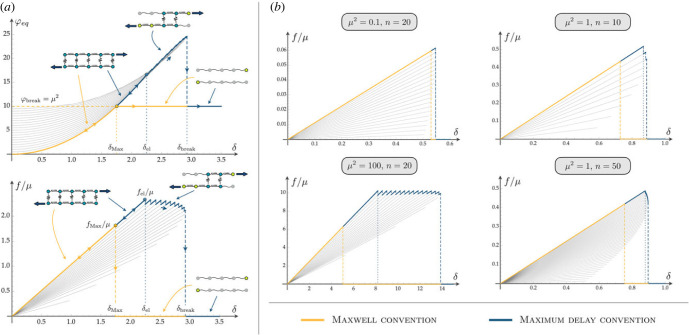
Melting transition behaviour under shear. (*a*) Equilibrium energy and force diagrams for a system with *μ*^2^ = 10, *n* = 20. Grey lines represent the equilibrium branches at different number of attached links *p*. Yellow lines represent the Maxwell convention, in which the global minima path is followed and the system jumps from a fully attached configuration to a fully detached configuration at *δ*_Max_. The blue path represents the maximum delay convention in which the system follows the equilibrium branches until they become unstable. In this case, a sequential detachment is attained starting from *δ*_el_ until the breaking threshold *δ*_break_ is reached. (*b*) Equilibrium force–displacement diagrams for different values of *μ*^2^ and *n*.

The evolution of the system in its wiggly energy landscape relies on the competition between loading rate and rate of overcoming energy barriers [6,52]. Three main time scales are involved: *τ*_int_, the time scale required for exploring the energy wells and relaxing to the local energy minima; *τ*_ext_, associated with the rate of external loading; *τ*_bar_, the time scale to overcome energy barriers and relax to the global energy minima. Here, we assume *τ*_int_ ≪ *τ*_bar_ and we consider two limiting cases. First, for high temperature and low rate of loading (*τ*_bar_ ≪ *τ*_ext_), the system configuration always corresponds to the global energy minima (*Maxwell convention*). Second, for low temperature and rate of loading (*τ*_int_ ≪ *τ*_ext_ ≪ *τ*_bar_), according with the *maximum delay convention* [43], we assume that the system remains in a metastable equilibrium branch (characterized by a certain value of *p*), i.e. in a local minimum of the energy, until it becomes unstable at *δ* = *δ*_max_(*p*). The corresponding equilibrium branches are represented by grey lines in figure 2.

Once again we remark that, based on previously reported energetic considerations, we restrict our analysis to single-block solutions with the purpose of obtaining clear analytical results. However, the following analysis can be adapted to the case with multiple blocks, where, again, the energetic competition is between the energy loss in fronts propagation against the elastic energy gain.

Under the Maxwell convention (figure 2), the system behaves cooperatively (for all values of *μ* and *n*) and follows elastically the fully attached branch *p* = *n* until at *δ*_Max_ it undergoes a transition to the fully detached state (yellow path in figure 2).

Under the maximum delay convention (blue lines in figure 2), the system switches from a given branch *p* = *q* to a branch with *p* = *q* − 1 unbroken bonds when *δ* = *δ*_max_(*q*). Interestingly, cooperativity is regulated by *μ* (see the right panel of figure 2). Decohesion is cooperative for low values of *μ* corresponding to high stiffness of the covalent versus non-covalent bonds. On the other hand, for large values of *μ*, after an elastic reversible regime (*δ* ≤ *δ*_el_, see figure 2), a ductile decohesion is observed, with a sawtooth force–displacement path representing the sequential phase-switching of the domains typically observed in the experiments [53]. As the assigned shear *δ* increases, the decohesion front coherently propagates until a second threshold *δ*_break_ is attained and the system fully detaches. Remarkably, as *μ* increases (ℓ_*o*_ ≪ *L*) the ductility of the system

**Figure 3.**
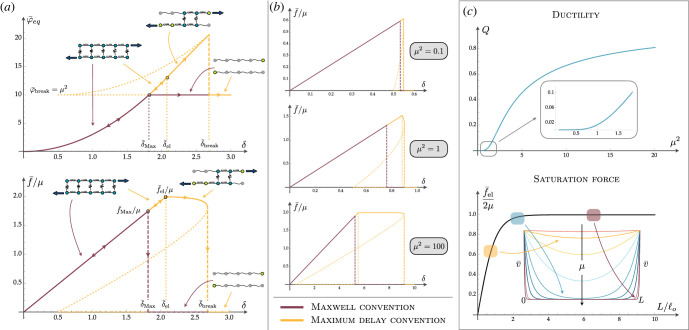
Continuum limit behaviour. (*a*) Equilibrium force and energy in the continuum limit when the Maxwell convention (brown line) and the maximum delay convention (yellow path) are considered. Also, in this case, we observe that in the first hypothesis the system behaves cooperatively, i.e. it switches from the fully attached to the fully detached configuration at ${\bar{\delta }}_{\mathrm{Max}}$, whereas in the maximum delay assumption a coherent front propagation is observed with a force plateau that depends on the cooperativity index *μ*. Here, we assume *μ*^2^ = 10. (*b*) Force–displacement diagram at different *μ*. The force plateau is larger for a larger value of *μ*. (*c*) Dissipative behaviour of the system in terms of ductility *Q*, which increases with *μ* and critical saturation force. For low *L*/ℓ_*o*_, the regime is linear (the shear $\bar{v}$ in the inset affects all the length of the chain) whereas for greater *L*/ℓ_*o*_ the force saturates because the shears are confined at the boundary of the system (see inset).

3.1 $$Q=\frac{\delta_{\mathrm{break}}-\delta_{\mathrm{el}}}{\delta_{\mathrm{el}}}$$

increases as its unfolding energy: $\int_{0}^{\Delta }f/(2\mu )$ (see figure 3*c*).

### 3.1. Continuum limit

With the purpose of analytical transparency, we also study the *continuum approximation*, relevant when the number of bonds is large. To determine this limit [54], we fix the contour length of the chain *L* = *n*ℓ, and we consider the limit of *n* → ∞ with ℓ → 0. To correctly rescale the mechanical quantities, we introduce the fraction of unbroken elements *π* = *p*/*n* ∈ (0, 1). Using classical arguments [55], the overall stiffness of the system reads

3.2 $${\bar{\kappa }}^{t}(\pi )\;:=\lim_{n\to +\infty }\kappa^{t}=\frac{4\mu }{\mu (2-\pi )+\text{coth}(\mu \pi )},$$

with force and energy given by (2.5) and (2.6).

Also in this limit, under the Maxwell convention the behaviour is always cooperative with an all-or-none transition at

3.3 $${\bar{\delta }}_{\mathrm{Max}}=\frac{1}{2}\sqrt{\mu^{2}+\mu \text{coth}\mu },$$

attained at a corresponding force of

3.4 $${\bar{f}}_{\mathrm{Max}}=\frac{2\mu^{2}}{\sqrt{\mu^{2}+\mu \text{coth}\mu }},$$

as described in figure 3*a*.

On the other hand, within the maximum delay convention, the cooperativity is regulated by *μ* and the elastic path holds until the first element breaks at

3.5 $${\bar{\delta }}_{\mathrm{el}}=\frac{1}{2}(1+\mu \text{tanh}\mu )$$

with a force

3.6 $${\bar{f}}_{\mathrm{el}}=2\mu \text{tanh}\mu \;\;$$

that corresponds to the maximum force attained by the system. This force combined with (2.10) and (2.3) determines how the critical decohesion force is regulated by the physical parameters

3.7 $${\bar{F}}_{c}=\sqrt{2k_{\tau }k_{e}}\text{tanh}\left(\sqrt{\frac{k_{\tau }}{2k_{e}}}\frac{L}{\Delta }\right).$$

It is important to remark that *μ* regulates the maximum force ${\bar{f}}_{\mathrm{el}}$ and controls the cooperativity through the microscopic mechanical properties. Indeed, *μ* can be varied either by changing the contour length *L*, or by changing ℓ_*o*_, which depends on bond stiffness. This reliance was already pointed out by de Gennes [33], who questioned the dependence of the rupture force of a double-stranded DNA (dsDNA) in terms of the number of base pairs. Conversely, when the length is fixed, we can study the force-varying bond properties. The dependence on *μ* of the cooperative/non-cooperative transition is crucial also in the limits

3.8 $$\begin{array}{rl} {\bar{F}}_{c}^{\mathrm{linear}} & \;:=\lim_{\mu \to 0}{\bar{F}}_{c}=2\mu^{2}\frac{k_{e}\Delta }{L}=\frac{k_{\tau }L}{\Delta },\\ {\bar{F}}_{c}^{\mathrm{plateau}} & \;:=\lim_{\mu \to \infty }{\bar{F}}_{c}=2\mu \frac{k_{e}\Delta }{L}=\sqrt{2k_{\tau }k_{e}}, \end{array}$$

obtaining a linear behaviour for small values of *μ* and a plateau for large values of *μ*.

In figure 3*a,b*, we summarize the influence of *μ* on the cooperativity and the stability properties of the melting transition. In particular, the ductility *Q* exhibits two regimes separated by the threshold ℓ_*o*_ ≃ *L* (*μ* ≃ 1). For smaller values of *μ* the behaviour is fragile. Indeed, in this case, the two chains are highly stiff, with a large value for the correlation length ℓ_*o*_. As described in the inset of the saturation force in figure 3*c*, in this regime, the stretch along all the attached bases in non-negligible (non-zero shears) and this leads to a sudden decohesion of all of them, resulting in an all-or-none melting transition. When *μ* is large, the behaviour is non-cooperative, with a ductile melting and a reserve after debonding begins. In the Introduction, we referred to this property as ‘stability’ of the melting transition. In this case, the correlation length is small and only a few bases have non-negligible stretch. This results in a coherent propagation front. In particular, this is reflected in the determined value of the saturation force as a function of *L*.

## 4. Comparison with existing experiments

We now analyse the ability of the proposed model in predicting quantitatively important phenomena regulating the melting transition of diverse biomolecules. We argue that this is possible because the cooperative and stability properties of the melting transition effects on which we focus in the following are mostly regulated by the interplay between the energy of local interactions and the decohesion energy of debonding fronts.

### 4.1. DNA

**Figure 4.**
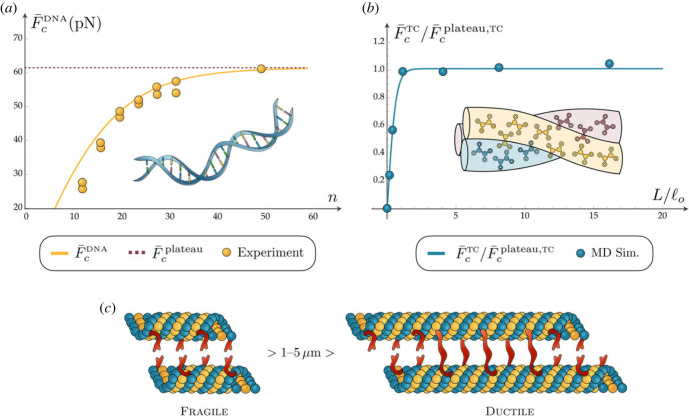
Comparison of our model with various examples of biological systems. (*a*) Saturation force for a DNA helix. (*b*) Saturation force with respect to the plateau force for a tropocollagen fibre. (*c*) Critical length separating a cooperative to a non-cooperative behaviour of a microtubule depending on its length.

First, we consider the melting transition of dsDNA in shear mode. The existence of a saturation force and of an internal length, corresponding to our localization length, is supported by both experiments [56] and theoretical results such as the coarse-grained model proposed in [34]. Of interest, de Gennes suggested the existence of a saturation number of base pairs, after which the DNA shear melting force saturates. More in detail, the experiments reported in figure 4*a* show the existence of an initial linear dependence of the force on the number of bases followed by a saturation effect, leading to a shear-lag type behaviour [57], in perfect accordance with our analytical results.

To show the possibility of applying our model in predicting actual experimental values, we determine the melting forces of dsDNA with lengths ranging from 12 to 50 base pairs [56]. We remark that the theory used in [56] is based on de Gennes’s approach [33] but, unfortunately, it requires unphysical values for some parameters. Here, to describe the energetic competition between inter- and intra-chains bonds we use well-established physical parameters [24,34, 58–63]. Specifically, since we focus on the decohesion in the double-stranded domain (attached block), the distance between bases can be taken to be ℓ^DNA^ = 3.4 Å and Δ^DNA^ = 0.025 nm (figure 4*a*). In this section, with a slight abuse of notation, we use indexes to refer mechanical quantities to the specific biological example.

To obtain the values of the stiffness, we compared the experimental value of the force plateau ${\bar{F\;}}_{c}^{\mathrm{plateau}}=61.5$ pN with the expression of ${\bar{F\;}}_{c}^{\mathrm{plateau}}$ in (3.8) to obtain $k{\;}_{\tau }^{\mathrm{DNA}}k_{e}^{\mathrm{DNA}}≃1890$ pN^2^. Then, by fitting the first three experimental values and comparing them with the expression of the linear force ${\bar{F}}_{c}^{\mathrm{linear}}$ in (3.8) we obtain $k_{\tau }^{\mathrm{DNA}}=0.24$ pN and $k_{e}^{\mathrm{DNA}}=7875$ pN. It is worth noticing that the obtained values are in perfect agreement with those deduced in [36] to fit the thermomechanical melting of DNA. As a result, we may now compute the cooperativity index *μ*^DNA^ ≃ 0.05*n* and the localization length $\ell_{o}^{\mathrm{DNA}}=6.5\;\mathrm{nm}$. Following, we predict a non-cooperative behaviour for *n* > *n*^DNA^ ≃ 20 in agreement with [34,56,61]. The comparison with the experimental unfolding forces at variable number of bases is shown in figure 4*a*. It is important to remark that despite the correct prediction of the critical value of the internal length ℓ_*o*_, other important features such as variations of the boundary conditions, three-dimensional effects, inhomogeneity of the bases, and the position of application of the force influence the DNA melting behaviour [64,65]. More sophisticated augmented models can be considered to describe such effects, but the physical clarity and analytical transparency of the obtained results justify the analysis of the considered prototypical system.

### 4.2. Tropocollagen fibres

Second, we determine the melting force for collagen fibrils forming tropocollagen molecules as a function of the molecule length. In this case, in accordance with our theoretical results, it is possible to distinguish two regimes as reported in [32]. For small molecular length (when compared with the localization length), the shear force is distributed along the whole molecule. The resulting melting energy thus increases linearly with the molecular length as exhibited by the molecular dynamic simulations reported in figure 4*b*. When the length is much larger, the force can be considered localized only in the external areas of the attached portion of the molecule and thus the melting force attains a saturation value, with no actual increase in molecule elongation. To reproduce the simulations reported in [32], we use again data from the literature and we follow the same procedure as above to obtain the values rescaled with the plateau force. As a result, we obtain the non-dimensional stiffnesses $k_{e}^{\mathrm{TC}}=0.3$, $k_{\tau }^{\mathrm{TC}}=1.7$. Then, we take known values of the decohesion elongation Δ^TC^ = 0.19 nm and the natural length ℓ^TC^ = 0.18 nm [32]. In figure 4*b*, we show the corresponding theoretical fitting of the data, which are in good agreement with molecular dynamics (MD) simulations.

### 4.3. Microtubules and tau proteins

Third, we consider the melting transition of tau proteins under shear. In particular, also in this case in accordance with our theoretical results, the experimental literature [66–71] indicates that short microtubules (1–5 μm) exhibit a fragile decohesion, whereas as length increases their transition becomes non-cooperative, with a sequential detachment of the tau proteins. This behaviour is of crucial importance for describing known effects on brain damage, possibly leading to neurodegenerative diseases [68]. Such an analysis will be proposed in our forthcoming work. The experimental behaviour is schematically reported in figure 4*c*. According to the experimental literature, in this case, we assume $k_{e}^{\mathrm{MT}}=1200$ MPa, $k_{\tau }^{\mathrm{MT}}=10$ MPa, Δ^MT^ = 65 nm and ℓ^MT^ = 30 nm, obtaining a cooperativity index *μ*^MT^ ≃ 0.03*n* and a localization length $\ell_{o}^{\mathrm{MT}}≃1\;\mu$, in agreement with the reported experimental values.

## 5. Discussion

Our theoretical results highlight the possible fundamental role of local interactions in cooperativity of several melting processes in double-stranded biomolecules. Under shear-type loading, we can describe different transition strategies. In the cooperative (fragile) regime, an all-or-none melting behaviour is attained, as in dsDNA [1]. On the other hand, in the non-cooperative regime, a propagating front is observed with a ductile transition [69,70]. Similar behaviour can be found in other contexts: double-stranded molecules under orthogonal loading [36]; decohesion in gecko-type adhesive systems under peeling [43]; in the field of force transmitted by single fibres in composite materials [12,57,72,73], indicating a saturation force and an internal length scale in accordance with the classical ‘shear lag theory’. The transition between these regimes is regulated by the introduced cooperativity index *μ* (figure 3) representing the main non-dimensional parameter of the paper.

We remark that in biological systems also thermal and rate effects can have a crucial role with the possibility of spontaneous transition at a bond stiffness-dependent critical temperature [6,36,74]. Specifically, for low rates thermal fluctuations can drive the system over the energy barriers of its wiggly energy landscape with unbinding forces smaller than the ones observed in the purely mechanical case. On the other hand, for higher rates the mechanical potential quickly overwhelms the most prominent energy barrier along the reaction pathway, leaving all states free to unbind [6,75–77]. These important effects will be the subject of further investigations by the authors of the present paper.

The main result of our model is that a non-cooperative→cooperative transition can be described as the result of purely local interactions. Previous models are based on the assumption of the important role of non-local interactions to obtain the same effects [39–41,78,79]. Hence, we have shown that cooperativity, melting stability and ductility may emerge from *local* mechanical interactions in double-stranded chains. Eventually, by considering a single parameter, it is possible to predict different melting strategies, also in agreement with experimental data and MD simulations, which in our opinion may be crucial both for the behaviour of many biological systems and in the design of new molecular-scale devices.

## Data Availability

All data used in this paper are extracted from the literature and cited. The data are provided in electronic supplementary material [80].
